# What predicts the knowledge of breastfeeding practices among late adolescent girls? evidence from a cross-sectional analysis

**DOI:** 10.1371/journal.pone.0258347

**Published:** 2021-10-08

**Authors:** Pradeep Kumar, Prem Shankar Mishra, Shobhit Srivastava, Debashree Sinha

**Affiliations:** 1 Department of Mathematical Demography & Statistics, International Institute for Population Sciences, Mumbai, Maharashtra, India; 2 Department of Population Research Centre, Institute for Social and Economic Change, Bengaluru, Karnataka, India; 3 Department of Development Studies, International Institute for Population Sciences, Mumbai, Maharashtra, India; Anglia Ruskin University, UNITED KINGDOM

## Abstract

**Introduction:**

Breastfeeding is one of the most effective ways to ensure infant health and survival. Inadequate breastfeeding practices, and knowledge among adolescent mothers have led to unprecedented infant and child morbidity and mortality. Given, the high global prevalence of adolescent mothers it is imperative to understand how the knowledge of breastfeeding practices operates among adolescent girls across different socio-economic settings.

**Materials & methods:**

Data was carried out from Understanding the Lives of Adolescents and Young Adults (UDAYA) survey, conducted in 2015–16. Descriptive statistics along with bivariate analysis was done to examine the preliminary results. For analysing the association between the binary outcome variable and other explanatory variables, binary logistic regression method was used. The explanatory variables were educational status of the respondent, media exposure, working status, ever pregnant status (only for married adolescent girls), sex and age of the household head, educational status of the head of the household, caste, religion, wealth index, residence and states.

**Results:**

About 42%, 50%, and 42% of married adolescent girls had knowledge of immediate breastfeeding, yellowish milk, and exclusive breastfeeding respectively. The odds of knowledge about immediate breastfeeding [married-AOR: 1.57; CI: 1.09–2.28 and unmarried-AOR: 1.30; CI: 1.08–1.55], yellowish milk feeding [married-AOR: 2.09; CI: 1.46–3.01 and unmarried-AOR: 1.39; CI: 1.17–1.66], and exclusive breastfeeding [married-AOR: 1.74; CI: 1.2–2.52 and unmarried-AOR: 1.46; CI: 1.22–1.76] were significantly more among adolescent girls aged 19 years old compared to 15 years old girls. Adolescent married and unmarried girls with 10 & above years of schooling were 1.82 times [AOR: 1.82; CI: 1.52–2.18] and 2.69 times [AOR: 2.69; CI: 2.08–3.47] more likely to have knowledge about immediate breastfeeding, 1.74 times [AOR: 1.74; CI: 1.45–2.09] and 2.10 times [AOR: 2.10; CI: 1.68–2.62] more likely to have knowledge about yellowish milk feeding, and 3.13 times [AOR: 3.13; CI: 2.6–3.78] and 3.87 times [AOR: 3.87; CI: 2.95–5.08] more likely to have knowledge about exclusive breastfeeding respectively than girls with no schooling.

**Conclusion:**

Breastfeeding practices and interpersonal counselling from elders in the household should be encouraged. Ongoing breastfeeding promotion programs of the government should promote high education of adolescent girls. Mass media interventions should be encouraged.

## Introduction

Breastfeeding is one of the most effective ways to ensure infant as well as child health and survival. Global estimates show that 1 in 3 infants under 6 months of age are exclusively breastfed i.e., only 44% of infants in 0–6 months of age are exclusively breastfed [1]. The World Health Organization (WHO) and United Nations Children’s Fund (UNICEF) have recommended exclusive breastfeeding from early initiation to the first six months of life and extended up to 24 months of age [2]. However, suboptimal breastfeeding or non-exclusive breastfeeding in the first six months of life has resulted in an increase in infant and child disease that has eventually led to death [2–4]. On one hand, while inadequate breastfeeding practices, knowledge, and attitude among mothers and specifically among adolescent mothers have led to unprecedented infant and child mortality and morbidity, on the other hand, shorter duration of breastfeeding practices have contributed to mothers’ health and well-being issues, like an increase in the risk of ovarian and breast cancer [1,3,5,6].

Still, the high global prevalence of adolescent motherhood is proving hard to shift in many low and middle-income countries [7]. A study at a global level shows that adolescent girls (aged 15–19 years) from 74 low-and middle-income countries across 28 years of period (1990–2018) do not have adequate educational and health support when they need it the most [8]. Furthermore, studies also demonstrate that socio-demographic disadvantage is a major driver of adolescent motherhood who struggles with severe problems in poor countries that require greater public policy attention and intervention [1,4,6,9–11]. Concerning socio-demographic and economic inequalities among adolescents also play a big part in early adolescent motherhood; at the same time, adolescent girls lack sufficient knowledge, practices, and interventions from the government and non-governmental organizations in dealing with the several infant and mothers’ health-related issues [2,12]. Therefore, in order to accelerate the progress towards meeting the United Nations Sustainable Development Goals (SDGs) on health and well-being of adolescent girls, a targeted approach is required where adolescent girls can receive a wide range of information related to sexual and reproductive health, infant breastfeeding, and child nutrition. In case of India, adolescent and young women living in the most disadvantageous conditions, for example those who are poor, have no education, live in rural and remote locations have the highest rates of adolescent motherhood and lack knowledge in several mother and child health-related practices [13–16]. Further, the lack of information on it also leads to several adverse health outcomes among mothers and children [6,10,12,17,18].

Knowledge associated with breastfeeding practices is most crucial for both infants and mothers. Factors that influence the knowledge of exclusive breastfeeding among adolescent girls vary across different sets of environments [8,9,19]. Further, sex of the head of household also influences breastfeeding practices. Socio-economic settings of adolescent girls also matter in the knowledge, attitude, and breastfeeding practices in low and middle-income countries [1,3,11,20–23]. Moreover, the factors that influence breastfeeding decisions in adolescent girls include social and cultural norms [8]. Personal beliefs about being a good mother are important to intention and initiation of breastfeeding [24]. In this way, the determinants of knowledge of breastfeeding practices among adolescent girls vary and it is affected by a wide range of socioeconomic, cultural, and individual factors [3,7,19]. Therefore, it becomes imperative to understand how the knowledge of breastfeeding practices operates among adolescent girls across different socio-economic settings. Despite its well-established benefits, breastfeeding among women is no longer a norm in many communities across countries, including India [6]. There are multi-factorial determinants of not providing exclusive breastfeeding and lack of adequate knowledge and practices at many levels [3,7,11,12]. Although there are multiple forms of breastfeeding like predominant, partial, non-breastfeeding, and exclusive breastfeeding which are prevailing all across the developed and developing countries [2], however, there are pieces of evidence that show that adolescent mothers do not follow appropriate breastfeeding norms [2,7,10]. This is true especially for adolescent mothers. There are huge differences in breastfeeding knowledge, attitude, and practices among mothers across different age groups [14,15,17,25,26]. Again, socio-cultural determinants among adolescent mothers also influence the knowledge of breastfeeding practices. Poor, uneducated, and lower community adolescent mothers are facing unprecedented knowledge gaps in the reproductive and child health care services compared to their counterparts [4,7,8,20,27]. Further, lack of appropriate and adequate knowledge of infant and young child feeding (IYCF) practices among adolescent mothers put their children at a heavy risk of several infectious diseases [28]. Furthermore, in developing countries like India, the proportion of adolescent girls (both married and unmarried) is quite high and lack of adequate information about appropriate breastfeeding practices among them have severely affected both mothers and children across different dimensions [4,13,19,29,30].

In this way, adequate knowledge of infant and young child feeding practices among adolescent girls can play a significant role in the health status and nutrition of children. The knowledge of breastfeeding practices also helps to enhance the immunity power of the infant and further controls anti-infectious diseases. Knowledge of exclusive breastfeeding practices has several benefits for both infants and mothers. However, despite strong evidence in support of exclusive breastfeeding, the prevailing knowledge and its prevalence have remained low among adolescent girls (both married and unmarried). Furthermore, Uttar Pradesh (19.3%) and Bihar (9.2%) contributes to the highest total adolescent population in the country [31]. In fact, 6.4% and 19.7% of girls aged 15–19 years are married in the states of Uttar Pradesh and Bihar respectively. Also, Bihar’s prevalence of child marriage is higher than the national average of 11.9% [32]. Since most of the child marriages result in teenage pregnancy, it is of utmost importance that we study this vulnerable sub-population of adolescent girls. Therefore, the objective of the paper is to understand the pathways in which different forms of knowledge, practices, and attitude predict breastfeeding practices among late adolescent girls in two big states of India i.e., Uttar Pradesh & Bihar. The hypothesis is that there are no predictors that influence breastfeeding practices, knowledge, and attitude among late-adolescent girls.

### Ethics statement

The ethical approval for this data was provided by the Population Council Institutional Review Board. Adolescents provided individual written consent to participate in the study, along with a parent/guardian for adolescents younger than 18. The Population Council identified referral services for counselling and health services to offer respondents if necessary, and fieldworkers were trained on ethical issues and sensitivity. In addition, interviewing boys and girls in separate segments helped minimize issues related to confidentiality and response bias.

## Materials and methods

### Data

Understanding the Lives of Adolescents and Young Adults (UDAYA) project survey data was used for this study. It is a cross-sectional survey conducted in two Indian states of Uttar Pradesh and Bihar, in 2016 by Population Council under the guidance of Ministry of Health and Family Welfare, Government of India. The UDAYA survey collected detailed information on family, media, community environment, assets acquired in adolescence, and quality of transitions to young adulthood indicators. The sample size for Uttar Pradesh and Bihar was 10,350 and 10,350 adolescents aged 10–19 years, respectively. The survey adopted a multi-stage systematic sampling design to provide the estimates for states as a whole as well as urban and rural areas of the states. The detailed information on the sampling procedure and survey design is published elsewhere [33]. The study used 5,206 married and 7,766 unmarried adolescent girls aged 15–19 years for the analysis.

### Variable description

#### Outcome variable

Three outcome variables were assessed through three different questions. 1. When should a new-born be offered breast milk? The responses were a. immediately (within one hour of birth) b. Other response c. don’t know. The variable was made binary by clubbing other responses and don’t know as 0 “don’t know” and 1 as “immediately”. 2. Should a new-born be fed the yellowish milk that comes from the mother’s breast after delivery? The responses were a. yes b. no and c. don’t know. The variable was coded into binary by clubbing no and don’t know as 0 “no” and “yes” as 1. 3. How long should a new-born be exclusively breastfed? The responses were a. six months b. other responses and c. don’t know. The variable was categorized as binary by clubbing other responses and don’t know as “don’t know” and “six months”. Therefore, the three outcome variables were 1. Immediate breastfeeding (don’t know and immediately) 2. Yellowish milk breastfeeds (no and yes) and 3. Exclusive breastfeeding (don’t know and six months).

#### Explanatory variable

The explanatory variables were selected based on extensive literature review [34–43].

1. Education of the respondent was coded as “no education”, “1–7 years”, “8–9 years” and “10 and above years”.
2. Media exposure was coded as “no”, “rare” and “frequent”.
3. Working status was coded as “no” and “yes”.
4. Ever pregnant status among married adolescent girls was coded as “no” and “yes”.
5. Sex and age of the household head were coded as “male and age less than 45 years”, “female and age less than 45 years”, “male and age more than equals to 45 years”, and “female and age more than equals to 45 years”.
6. Education of the head of the household was coded as “no education”, “1–7 years”, “8–10 years” and “11 and above years”.
7. Caste was coded as “Scheduled caste/Scheduled Tribe (SC/ST)” and “non-SC/ST”.
8. Religion was coded as “Hindu” and “non-Hindu”.
9. Wealth index was coded as “poorest” “poorer” middle” richer” and richest”.
10. Residence was available in the data as “urban” and “rural”.
11. Survey was conducted in two states “Uttar Pradesh” and “Bihar”.

### Statistical analysis

Descriptive statistics along with bivariate analysis was done to examine the preliminary results. Chi-square test was used to test the level of significance during the bivariate analysis. P-values were stated in the tables. For analysing the association between the binary outcome variable and other explanatory variables binary logistic regression method was used. The outcome variable was knowledge about breastfeeding practices among adolescent unmarried and married girls aged 15–19 years. We termed adolescent girls aged 15–19 as late adolescents. Adjusted odds ratio (AOR) was presented in the tables at 95% confidence interval (CI). The estimates were presented at p<0.05.

The equation for logistic distribution is

$$ln\left(\frac{\pi }{1-\pi }\right)=\alpha +\beta_{1}X_{1}+\beta_{2}X_{2}+\beta_{3}X_{3}\ldots \ldots .\beta_{n}X_{n}$$

Where *β*_0_,…..,*β_M_* is regression coefficient indicating the relative effect of a particular explanatory variable on the outcome. These coefficients change as per the context in the analysis in the study. Before putting explanatory variables in the multivariable binary logistic regression analysis, variance inflation factor (VIF) was estimated and it was found that there was no multi-collinearity between the explanatory variables. Additionally, *svyset* command was used in STATA 13 to adjust the analysis for complex survey design and individual weights were used to make the estimates representative. The present analysis was done using STATA 13.

## Results

Socio-demographic characteristics of the study population are presented in *Table 1*. About 32 per cent and 42 per cent of married adolescent girls were 18 and 19 years old respectively whereas only 18 per cent and 11 per cent of unmarried girls belonged to the same age group. Nearly one-fourth of married girls had 8–9 or 10 & above years of schooling; moreover 42 per cent of unmarried girls had 10 & above years of schooling. Around 42 per cent of married and 59 per cent of unmarried girls had frequent media exposure, 12 per cent married and 22 per cent of unmarried girls were working, and about 55 per cent of married girls were ever pregnant. A higher percentage of males and age ≥45 years were head of the household (married-56% and unmarried-61%) and majority of the head of the households had no education.

**Table 1:** Socio-demographic characteristics of late adolescent girls.

Background characteristics	Married		Unmarried	
	Sample	Percentage	Sample	Percentage
**Age (in years)**				
15	130	2.5	1,944	25.0
16	378	7.3	1,932	24.9
17	819	15.7	1,578	20.3
18	1,677	32.2	1,402	18.1
19	2,202	42.3	910	11.7
**Education (in years)**				
No Schooling	1,251	24.0	571	7.4
1–7	1,171	22.5	1,459	18.8
8–9	1,379	26.5	2,436	31.4
10 and above	1,404	27.0	3,300	42.5
**Media exposure**				
No exposure	1,358	26.1	1,096	14.1
Rare	1,673	32.1	2,093	27.0
Frequent	2,175	41.8	4,577	58.9
**Working status**				
No	4,596	88.3	6,071	78.2
Yes	610	11.7	1,695	21.8
**Ever pregnant**				
No	2,330	44.8	N. A.	N. A.
Yes	2,876	55.2	N. A.	N. A.
**Sex and age of the head**				
Male and <45 years	1,650	31.7	2,095	27.0
Female and <45 years	258	5.0	456	5.9
Male and ≥45 years	2,920	56.1	4,769	61.4
Female and ≥45 years	379	7.3	446	5.7
**Education of the head (in years)**				
No schooling	3,180	61.1	3,250	41.9
1–7	740	14.2	1,280	16.5
8–10	982	18.9	2,054	26.5
11 and above	303	5.8	1,183	15.2
**Wealth index**				
Poorest	759	14.6	938	12.1
Poorer	1,069	20.5	1,339	17.2
Middle	1,222	23.5	1,634	21.0
Richer	1,262	24.2	1,918	24.7
Richest	895	17.2	1,938	25.0
**Caste**				
Scheduled Caste/Scheduled Tribe	1,543	29.7	1,819	23.4
Non- Scheduled Caste/Scheduled Tribe	3,663	70.4	5,947	76.6
**Religion**				
Hindu	4,306	82.7	5,943	76.5
Non-Hindu	900	17.3	1,823	23.5
**Residence**				
Urban	730	14.0	1,344	17.3
Rural	4,476	86.0	6,422	82.7
**State**				
Uttar Pradesh	3,218	61.8	5,562	71.6
Bihar	1,988	38.2	2,204	28.4
**Total**	5,206	100.0	7,766	100.0

**Fig 1:**
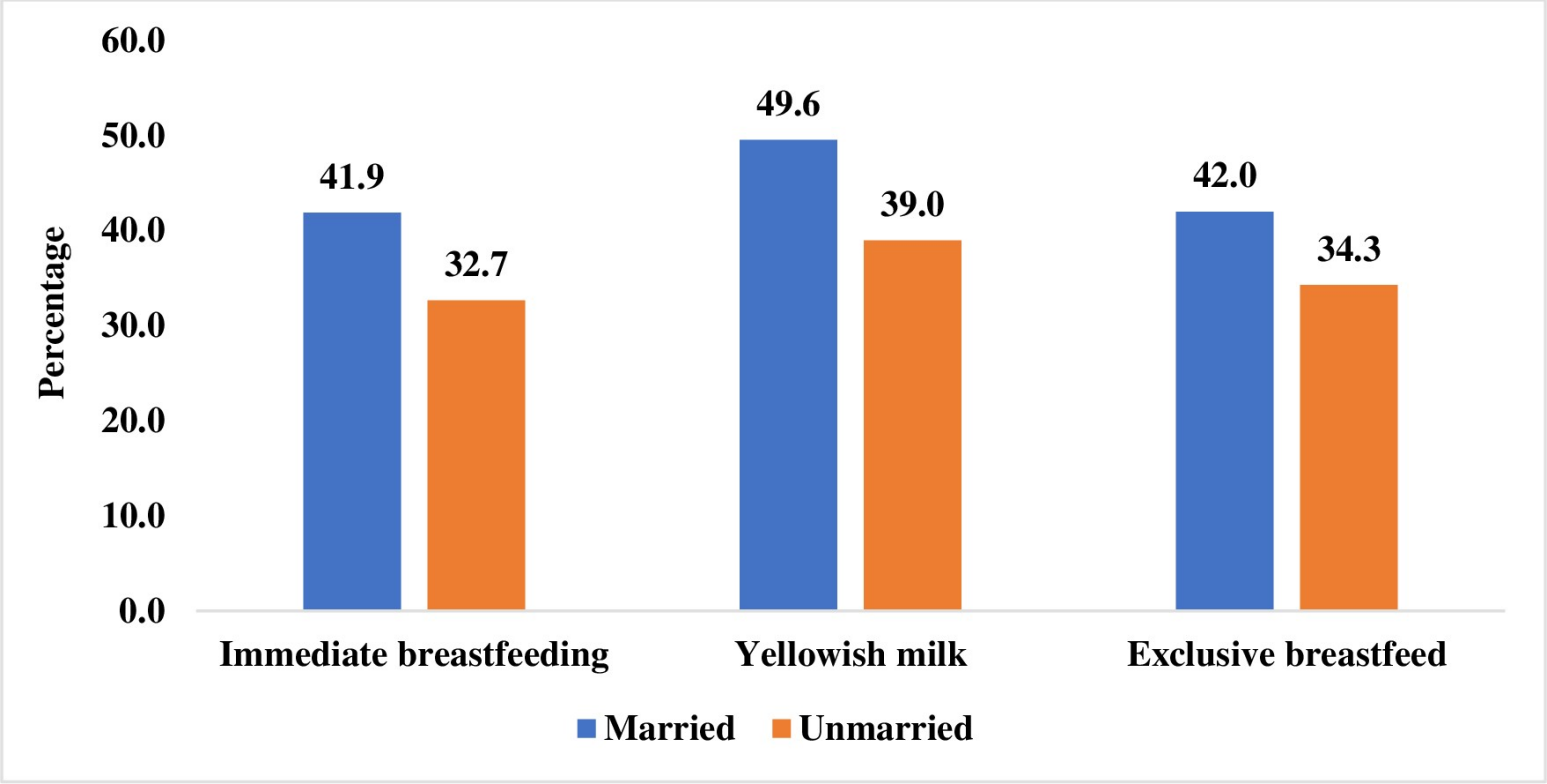
Knowledge of various breastfeeding practices among married and unmarried adolescent girls aged 15–19 years.

Fig 1 depicts that knowledge of breastfeeding practices was higher among married adolescent girls than unmarried ones. For instance, about 42 per cent, 50 per cent, and 42 per cent of married adolescent girls had knowledge of immediate breastfeeding, yellowish milk, and exclusive breastfeeding respectively.

Knowledge about immediate breastfeeding among late adolescent girls is shown in *Table 2A*. Age and education of adolescent girls had a positive association with knowledge of immediate breastfeeding. In other words, knowledge about immediate breastfeeding increased with the increase of age and education of married and unmarried girls. The knowledge about immediate breastfeeding was significantly higher among girls who had frequent media exposure (married- 45.3% and unmarried-39.3%) and those whose household head had 11 & above years of schooling (married-48.8% and unmarried-41.7%). Similarly, Hindu adolescent girls (married-43.2% and unmarried-34.1%) and girls who lived in urban areas (married-45.3% and unmarried-38.1%) had more knowledge about immediate breastfeeding.

**Table 2:** a Percentage distribution for knowledge about immediate breastfeeding among late adolescent girls by background characteristics.

Background characteristics	Married		Unmarried	
	%	p-value	%	p-value
**Age (in years)**		<0.001		<0.001
15	30.8		27.6	
16	33.6		31.0	
17	38.8		34.2	
18	40.3		35.9	
19	46.3		39.3	
**Education (in years)**		<0.001		<0.001
No Schooling	35.0		15.2	
1–7	37.8		22.6	
8–9	43.7		30.7	
10 and above	49.7		41.6	
**Media exposure**		<0.001		<0.001
No exposure	37.8		17.8	
Rare	40.8		26.0	
Frequent	45.3		39.3	
**Working status**		<0.001		<0.001
No	42.4		34.1	
Yes	37.7		27.7	
**Ever pregnant**		<0.001		
No	36.3		N. A.	
Yes	46.4		N. A.	
**Sex and age of the head**		0.261		<0.001
Male and <45 years	41.2		31.1	
Female and <45 years	41.4		27.8	
Male and ≥45 years	42.8		33.7	
Female and ≥45 years	38.6		33.9	
**Education of the head (in years)**		<0.001		<0.001
No schooling	39.2		27.4	
1–7	44.1		32.6	
8–10	46.0		35.9	
11 and above	48.8		41.7	
**Wealth index**		<0.001		<0.001
Poorest	36.9		26.6	
Poorer	37.1		25.9	
Middle	45.6		28.5	
Richer	45.1		35.3	
Richest	42.4		41.2	
**Caste**		0.713		0.592
Scheduled Caste/Scheduled Tribe	43.7		32.4	
Non- Scheduled Caste/Scheduled Tribe	41.1		32.8	
**Religion**		0.017		<0.001
Hindu	43.2		34.1	
Non-Hindu	35.5		28.1	
**Residence**		<0.001		<0.001
Urban	45.3		38.1	
Rural	41.3		31.5	
**State**		0.122		0.090
Uttar Pradesh	41.4		33.0	
Bihar	42.7		31.9	

%: Percentage; p-value based on chi-square test.

**Table 2:** b Percentage distribution for knowledge about yellowish milk breastfeeding among late adolescent girls by background characteristics.

Background characteristics	Married		Unmarried	
	%	p-value	%	p-value
**Age (in years)**		<0.001		<0.001
15	31.3		32.5	
16	35.9		35.3	
17	46.8		40.7	
18	47.6		43.5	
19	55.5		50.9	
**Education (in years)**		<0.001		<0.001
No Schooling	43.4		25.6	
1–7	43.0		28.6	
8–9	49.7		32.9	
10 and above	60.3		50.4	
**Media exposure**		<0.001		<0.001
No exposure	44.9		24.5	
Rare	47.5		29.6	
Frequent	54.1		46.8	
**Working status**		0.384		0.007
No	49.9		39.6	
Yes	46.8		37.0	
**Ever pregnant**		0.001		
No	40.5		N.A.	
Yes	56.9		N.A.	
**Sex and age of the head**		0.114		0.536
Male and <45 years	51.2		38.0	
Female and <45 years	44.3		36.8	
Male and ≥45 years	49.5		40.0	
Female and ≥45 years	46.6		35.1	
**Education of the head (in years)**		<0.001		<0.001
No schooling	46.7		33.3	
1–7	51.6		34.6	
8–10	52.8		42.1	
11 and above	62.1		53.6	
**Wealth index**		<0.001		<0.001
Poorest	46.0		30.6	
Poorer	44.6		28.2	
Middle	48.2		35.6	
Richer	52.9		43.2	
Richest	55.7		49.3	
**Caste**		0.984		0.464
Scheduled Caste/Scheduled Tribe	50.9		37.4	
Non- Scheduled Caste/Scheduled Tribe	49.0		39.5	
**Religion**		0.396		<0.001
Hindu	50.4		40.4	
Non-Hindu	45.4		34.4	
**Residence**				
Urban	57.4		48.9	
Rural	48.3		36.9	
**State**		0.042		<0.001
Uttar Pradesh	49.2		39.1	
Bihar	50.2		38.8	

%: Percentage; p-value based on chi-square test.

**Table 2:** c Percentage distribution for knowledge about exclusive breastfeeding among late adolescent girls by background characteristics.

Background characteristics	Married		Unmarried	
	%	p-value	%	p-value
**Age (in years)**		<0.001		<0.001
15	31.6		28.0	
16	26.7		30.5	
17	36.7		38.3	
18	41.1		38.1	
19	47.9		42.9	
**Education (in years)**		<0.001		<0.001
No Schooling	27.6		13.2	
1–7	33.5		19.7	
8–9	44.6		29.2	
10 and above	59.4		48.2	
**Media exposure**		<0.001		<0.001
No exposure	30.0		16.4	
Rare	41.9		24.5	
Frequent	49.6		43.0	
**Working status**		<0.001		<0.001
No	43.0		37.1	
Yes	34.2		24.3	
**Ever pregnant**		<0.001		
No	36.3		N. A.	
Yes	46.7		N. A.	
**Sex and age of the head**		<0.001		0.174
Male and <45 years	40.0		34.7	
Female and <45 years	45.2		34.3	
Male and ≥45 years	43.8		34.6	
Female and ≥45 years	34.7		29.4	
**Education of the head (in years)**		<0.001		<0.001
No schooling	40.4		27.0	
1–7	41.0		32.9	
8–10	46.6		37.7	
11 and above	59.1		49.8	
**Wealth index**		<0.001		<0.001
Poorest	34.2		20.8	
Poorer	33.2		23.6	
Middle	41.2		31.5	
Richer	47.0		37.8	
Richest	53.2		47.0	
**Caste**		<0.001		<0.001
Scheduled Caste/Scheduled Tribe	37.5		30.5	
Non- Scheduled Caste/Scheduled Tribe	43.9		35.5	
**Religion**		0.014		<0.001
Hindu	43.0		37.1	
Non-Hindu	37.5		25.2	
**Residence**		<0.001		<0.001
Urban	46.8		42.6	
Rural	41.2		32.5	
**State**		<0.001		<0.001
Uttar Pradesh	38.3		31.6	
Bihar	48.0		41.2	

%: Percentage; *p-value based on chi-square test.

*Table 2B* shows knowledge about yellowish milk breastfeeding among married and unmarried adolescent girls. It was found that knowledge about yellowish milk breastfeeding was significantly higher among adolescent girls aged 19 years and who had 10 & above years of schooling irrespective of their marital status. Moreover, media exposure had a positive relationship with knowledge about yellowish milk breastfeeding. This knowledge was lowest among girls whose households’ heads had no schooling (married-46.7% and unmarried-33.3%) and belonged to poorer wealth index (married-44.6% and unmarried-28.2%). Though, it was significantly higher among those whose household heads had 11 & above years of schooling and belonged to the richest families. The knowledge about yellowish milk breastfeeding was significantly higher among Hindu girls (married-50.4% and unmarried-40.4%) and those who lived in urban areas (married-57.4% and unmarried-48.9%).

*Table 2C* provides the percentage of adolescent married and unmarried girls who had knowledge about exclusive breastfeeding. It was found that adolescent girls aged 19 years (married-47.9% and unmarried-42.9%) and those who had 10 & above years of schooling (married-59.4% and unmarried-48.2%) reported significantly higher knowledge about exclusive breastfeeding. Moreover, the knowledge about exclusive breastfeeding was lowest among adolescent girls who had no media exposure and it was highest among those who had frequent media exposure irrespective of their marital status. Interestingly, not working girls (married-43% and unmarried-37.1%) had significantly higher knowledge than working girls. Household heads’ education had a significant positive association with the knowledge about exclusive breastfeeding. Adolescent girls who belonged to the richest families (married-53.2% and unmarried-47%) and non-SC/ST caste group (married-43.9% and unmarried-35.5%) had significantly higher knowledge about exclusive breastfeeding. Similarly, Hindu girls (married-43% and unmarried-7.1%) and those who lived in urban areas (married-46.8% and unmarried-42.6%) had significantly higher knowledge about exclusive breastfeeding compared to their counterparts.

Estimates from logistic regression analysis for knowledge about various breastfeeding practices among adolescent girls are presented in *Table 3*. It was found that the odds of knowledge about immediate breastfeeding [married-AOR: 1.57; CI: 1.09–2.28 and unmarried-AOR: 1.30; CI: 1.08–1.55], yellowish milk feeding [married-AOR: 2.09; CI: 1.46–3.01 and unmarried-AOR: 1.39; CI: 1.17–1.66], and exclusive breastfeeding [married-AOR: 1.74; CI: 1.2–2.52 and unmarried-AOR: 1.46; CI: 1.22–1.76] was significantly more likely among adolescent girls aged 19 years old compared to 15 years old girls. Similarly, adolescent married and unmarried girls with 10 & above years of schooling were 1.82 times [AOR: 1.82; CI: 1.52–2.18] and 2.69 times [AOR: 2.69; CI: 2.08–3.47] more likely to have knowledge about immediate breastfeeding, 1.74 times [AOR: 1.74; CI: 1.45–2.09] and 2.10 times [AOR: 2.10; CI: 1.68–2.62] more likely to have knowledge about yellowish milk feeding, and 3.13 times [AOR: 3.13; CI: 2.6–3.78] and 3.87 times [AOR: 3.87; CI: 2.95–5.08] more likely to have knowledge about exclusive breastfeeding respectively than girls with no schooling. Moreover, the likelihood of knowledge about immediate breastfeeding [married-AOR: 1.22; CI: 1.03–1.45 and unmarried-AOR: 1.80; CI: 1.46–2.21], yellowish milk feeding [married-AOR: 1.25; CI: 1.05–1.48 and unmarried-AOR: 1.90; CI: 1.57–2.29] and exclusive breastfeeding [married-AOR: 1.72; CI: 1.44–2.05 and unmarried-AOR: 2.07; CI: 1.67–2.57] was significantly more likely among adolescent girls who had frequent media exposure compared to those who had no media exposure. As expected, the odds of knowledge about immediate breastfeeding [AOR: 1.73; CI: 1.53–1.96], yellowish milk breastfeeding [AOR: 1.82; CI: 1.61–2.06], and exclusive breastfeeding [AOR: 1.67; CI: 1.47–1.90] was 73 per cent, 82 per cent, and 67 per cent significantly more likely among ever pregnant married adolescent girls than those who were not ever pregnant.

**Table 3:** Logistic regression estimates for knowledge about various breastfeeding practices among late adolescent girls by background characteristics.

Background characteristics	Immediate Breastfeeding		Yellowish milk		Exclusive breastfeeding	
	Married	Unmarried	Married	Unmarried	Married	Unmarried
	AOR (95% CI)	AOR (95% CI)	AOR (95% CI)	AOR (95% CI)	AOR (95% CI)	AOR (95% CI)
**Age (in years)**						
15	Ref.	Ref.	Ref.	Ref.	Ref.	Ref.
16	0.97(0.65,1.45)	1.0(0.86,1.15)	1.15(0.78,1.71)	1.09(0.95,1.25)	0.85(0.57,1.28)	1.08(0.93,1.25)
17	1.24(0.85,1.81)	1.07(0.92,1.25)	1.59*(1.1,2.31)	1.13(0.97,1.31)	1.17(0.8,1.71)	1.28*(1.09,1.5)
18	1.35(0.93,1.95)	1.30*(1.11,1.53)	1.61*(1.13,2.32)	1.30*(1.11,1.52)	1.28(0.88,1.85)	1.36*(1.16,1.6)
19	1.57*(1.09,2.28)	1.30*(1.08,1.55)	2.09*(1.46,3.01)	1.39*(1.17,1.66)	1.74*(1.2,2.52)	1.46*(1.22,1.76)
**Education (in years)**						
No Schooling	Ref.	Ref.	Ref.	Ref.	Ref.	Ref.
1–7	1.14(0.97,1.35)	1.46*(1.12,1.9)	1(0.85,1.17)	1.15(0.92,1.45)	1.17(0.98,1.39)	1.64*(1.23,2.18)
8–9	1.32*(1.11,1.56)	2.03*(1.58,2.62)	1.14(0.97,1.35)	1.25*(1,1.55)	1.89*(1.59,2.24)	2.32*(1.77,3.04)
10 and above	1.82*(1.52,2.18)	2.69*(2.08,3.47)	1.74*(1.45,2.09)	2.10*(1.68,2.62)	3.13*(2.6,3.78)	3.87*(2.95,5.08)
**Media exposure**						
No exposure	Ref.	Ref.	Ref.	Ref.	Ref.	Ref.
Rare	1.06(0.9,1.24)	1.26*(1.02,1.56)	1.08(0.92,1.26)	1.06(0.87,1.29)	1.23*(1.04,1.45)	1.26*(1.01,1.58)
Frequent	1.22*(1.03,1.45)	1.80*(1.46,2.21)	1.25*(1.05,1.48)	1.90*(1.57,2.29)	1.72*(1.44,2.05)	2.07*(1.67,2.57)
**Working status**						
No	Ref.	Ref.	Ref.	Ref.	Ref.	Ref.
Yes	0.89(0.74,1.08)	0.87*(0.76,0.99)	1.12(0.93,1.34)	1.09(0.96,1.24)	1.0(0.82,1.21)	0.93(0.81,1.07)
**Ever pregnant**						
No	Ref.		Ref.		Ref.	
Yes	1.73*(1.53,1.96)		1.82*(1.61,2.06)		1.67*(1.47,1.90)	
**Sex and age of the head**						
Male and <45 years	Ref.	Ref.	Ref.	Ref.	Ref.	Ref.
Female and <45 years	1.18(0.9,1.54)	0.96(0.76,1.2)	1.08(0.83,1.41)	1.15(0.93,1.41)	1.28(0.97,1.68)	1.04(0.84,1.3)
Male and ≥45 years	1.02(0.9,1.17)	1.12(0.99,1.25)	0.93(0.81,1.06)	1.01(0.91,1.13)	1.06(0.92,1.21)	0.99(0.88,1.11)
Female and ≥45 years	0.86(0.67,1.1)	1.37*(1.1,1.7)	0.82(0.64,1.05)	1.04(0.84,1.29)	0.67*(0.52,0.87)	0.92(0.73,1.15)
**Education of the head (in years)**						
No schooling	Ref.	Ref.	Ref.	Ref.	Ref.	Ref.
1–7	1.14(0.97,1.35)	1.06(0.92,1.23)	0.99(0.84,1.17)	0.93(0.81,1.07)	0.99(0.84,1.18)	1.04(0.9,1.21)
8–10	1.18*(1.01,1.37)	1.04(0.91,1.19)	1.08(0.92,1.26)	1.06(0.93,1.21)	1.06(0.9,1.24)	1.05(0.92,1.21)
11 and above	1.02(0.8,1.31)	1.24*(1.06,1.46)	1.30(1,1.68)	1.36*(1.16,1.6)	1.09(0.84,1.42)	1.19*(1.01,1.39)
**Wealth index**						
Poorest	Ref.	Ref.	Ref.	Ref.	Ref.	Ref.
Poorer	0.94(0.77,1.15)	0.94(0.75,1.17)	1.02(0.84,1.24)	0.82(0.66,1.01)	0.9(0.74,1.1)	1.01(0.8,1.29)
Middle	1.05(0.87,1.28)	0.89(0.72,1.1)	0.99(0.82,1.21)	0.87(0.72,1.07)	1.04(0.85,1.27)	1.19(0.95,1.49)
Richer	1.05(0.85,1.3)	0.98(0.8,1.22)	1.1(0.9,1.36)	1.04(0.85,1.27)	1.14(0.92,1.42)	1.43*(1.14,1.79)
Richest	1.02(0.79,1.32)	1.06(0.85,1.33)	1.26(0.98,1.62)	1.07(0.86,1.32)	1.38*(1.06,1.8)	1.64*(1.29,2.07)
**Caste**						
Scheduled Caste/Scheduled Tribe	Ref.	Ref.	Ref.	Ref.	Ref.	Ref.
Non- Scheduled Caste/Scheduled Tribe	0.98(0.85,1.12)	0.86*(0.76,0.98)	0.95(0.83,1.08)	0.87*(0.76,0.98)	1.07(0.93,1.23)	0.97(0.85,1.11)
**Religion**						
Hindu	Ref.	Ref.	Ref.	Ref.	Ref.	Ref.
Non-Hindu	0.86(0.73,1.02)	0.94(0.83,1.07)	0.96(0.82,1.14)	0.84*(0.75,0.95)	0.94(0.79,1.12)	0.76*(0.67,0.86)
**Residence**						
Urban	Ref.	Ref.	Ref.	Ref.	Ref.	Ref.
Rural	0.89(0.78,1.02)	0.97(0.87,1.08)	0.80*(0.71,0.92)	0.78*(0.7,0.86)	0.96(0.83,1.09)	0.89*(0.8,1)
**State**						
Uttar Pradesh	Ref.	Ref.	Ref.	Ref.	Ref.	Ref.
Bihar	1.13(1,1.28)	1.13*(1.02,1.25)	1.21*(1.07,1.38)	1.39*(1.25,1.53)	1.98*(1.73,2.27)	1.88*(1.7,2.08)

*p<0.05; AOR: Adjusted odds ratio; CI: Confidence interval; Ref. reference.

## Discussion

Breastfeeding is one of the most effective method of infant feeding to meet its nutritional, metabolic, and psychological needs [44]. A recent study highlights the importance of breastfeeding in preventing diarrhoea, and acute respiratory infection among children aged 0–23 years [45]. Yet, the practice of breastfeeding is associated with various myths and superstitions [46]. Thus, in the present study, we analyse data from Understanding the Lives of Adolescents and Young Adults (UDAYA) to explore the background characteristics that determines an unmarried as well as a married adolescent’s knowledge, and attitude for breastfeeding practices in the states of Bihar and Uttar Pradesh in India. The study found that knowledge of breastfeeding practices was higher among married adolescent girls than unmarried ones. Similarly, both the bivariate and multivariate analysis indicated that the knowledge about immediate breastfeeding, yellowish milk breastfeeding, and exclusive breastfeeding was more among those adolescents (both married and unmarried) whose age was 19 years, had more than 10 years or above education, and was frequently exposed to media. Moreover, married adolescents who were ever pregnant were more likely to have knowledge about all the breastfeeding practices.

The literature emphasizes a close and positive association of mother’s marital status, age, and education with knowledge about breastfeeding practices [47–49]. Greater maternal age is indicative that the mother is experienced, aware of the benefits of breastfeeding and also practices it. Similarly, high education is more accurate, informative and useful to understand the patterns of breastfeeding because more educated mothers might be up to date with recommendations made by health authorities [50]. A study analysing data from the Nepal Demographic and Health Surveys showed that maternal education was associated with a higher likelihood of early initiation of breastfeeding. The same study suggested that while in the long-term approaches to prioritise education for women and girls should be taken up, however in the short-term uneducated mothers should be targeted with breastfeeding promotion strategies such as counselling and peer education [51]. A descriptive cross-sectional study conducted in Bangladesh illustrated that father’s education was as important as the mother’s education in having significant knowledge about breastfeeding practices [52].

The role of mass media in promoting various health and developmental programs cannot be ignored. The results of the present study also depict a similar pattern i.e., adolescent girls (both married and unmarried) who were exposed to mass media had a higher likelihood of knowledge about various breastfeeding practices. This finding is similar to the results of other studies conducted in developing countries. For instance, though no improvements in breastfeeding practices were reported, the proportion of mothers who received breastfeeding information via television increased after a media campaign on breastfeeding in Sindh Province, Pakistan was introduced [53]. In another study from rural Bangladesh, a national mass media campaign was launched using television channels to improve poor IYCF (Infant and Young Children Feeding) practices. It was observed that both the knowledge and practice of breastfeeding had improved significantly [54]. Again, in Vietnam, exposure to both mass-media and interpersonal counselling was associated with greater knowledge, intention, beliefs, social norms and self-efficacy about exclusive breastfeeding than exposure to either or none of these interventions [55].

## Strengths and limitations

The study highlights the importance of background characteristics in determining the knowledge of breastfeeding practices among late adolescent girls. However, the present study has some limitations too. First, cautious interpretation is required when generalizing the results for India because analysis is based only on two states of India, i.e., Uttar Pradesh & Bihar. Secondly, causal inference between the three outcome variables and independent variables are limited due to the use of cross-sectional data.

## Conclusion

One of the most important intervention required for child survival is encouraging breastfeeding practices. Hence, to ensure that adequate awareness is generated among the masses, especially to mothers on the benefits of breastfeeding, the Ministry of Health & Family Welfare launched National Breastfeeding Promotion Programme- MAA (Mothers’ Absolute Affection). However, when the mother is an adolescent the risk of proper knowledge and practice of breastfeeding increases. Evidence from the present study highlighted the importance of the mother’s high education, older age and frequent exposure to mass media as important determinants of knowledge about breastfeeding. The ongoing breastfeeding promotion programs can emphasize these determinants to improve the existing knowledge about breastfeeding practices. Moreover, breastfeeding practices and interpersonal counselling either from peers or elders in the household should be encouraged, given the young age of the mothers. Again, although the legal age of marriage for females in India is 18 years, it is shocking to learn that not only adolescent girls are married off at the age of 15 years but are also pregnant. This practice should be discouraged as much as possible. Furthermore, mass media interventions should be encouraged.
